# Emotional facial expression in women recovered from anorexia nervosa

**DOI:** 10.1186/1471-244X-13-291

**Published:** 2013-11-07

**Authors:** Helen Davies, Ulrike Schmidt, Kate Tchanturia

**Affiliations:** 1 Division of Psychological Medicine, King’s College London, Institute of Psychiatry, De Crespigny Park, PO59, London SE5 8AF, UK

**Keywords:** Anorexia, Recovered, Emotion, Facial expression, Eating disorders

## Abstract

**Background:**

Recent models of anorexia nervosa (AN) have emphasised the importance of social and emotional difficulties as maintenance factors of the disorder, however, empirical data are limited. The aim of this study was to examine whether altered emotional facial expression, previously observed in people currently ill with anorexia nervosa, is limited to the ill state or present in people recovered from the illness.

**Methods:**

The sample consisted of 123 participants [49 AN, 21 recovered AN (RecAN) and 53 healthy controls (HC)]. Participants watched three films clips (amusing, neutral, sad) whilst their facial expressions were recorded and completed the positive and negative affect scale (PANAS) to record subjective experience. Facial expressions were subsequently coded for frequency of positive and negative expression and frequency of looking away.

**Results:**

In response to the amusing clip, AN participants showed significantly less positive expression than both HC and RecAN groups and both AN and RecAN showed more negative expression than HC with no difference between groups in looking away.

In response to the sad clip there was no difference between groups in positive expression, but current AN participants showed significantly less negative expression than HC and looked away from the stimuli more than RecAN or HC.

In terms of their subjective emotional experience, patients with current AN reported less positive emotion in response to both the amusing and the sad film clip. There was no difference between groups in subjective negative experience.

**Conclusions:**

Alterations in facial expression are present in people currently ill with AN contributing to the social difficulties found in AN and potentially exacerbating resistance to treatment. Some alterations in facial expression are found in women with a past history of AN but not to the same extent as those shown in the currently ill group. Future studies need to use a wider range of stimuli involving different emotions to corroborate findings.

## Background

Anorexia nervosa (AN) is a disorder with major psychological, physical and social sequelae [1] as well as high mortality [2]. However, progress in the treatment of adults with AN remains limited [3,4]. Recent maintenance models [5], supported by experimental and self-report studies [6-9], highlight significant and wide ranging impairments in the socio-emotional domain in people currently ill with AN. Such maintaining factors may contribute to the resistance to treatment. For example, there is altered emotional facial expression [8], which can impact on effective emotion regulation and social interaction [10-12]. A major problem, however, with undertaking research in patients currently ill with AN, is the confounding effect of starvation, and thus low body mass index (BMI), on potential aetiological factors, such as altered emotional expression. For example, starvation in healthy people has been shown to be associated with various socio-emotional related changes [13].

As a first step in disentangling state and trait factors, cross sectional comparisons of ill and recovered patients can be of use. This is the strategy employed here to investigate whether altered emotional facial expression [8] is present in people with a past history of AN. Studies of facial expression in other clinical groups [schizophrenia and post traumatic stress disorder (PTSD)] of people who are in remission [14,15] or who are at risk of mental illness [16] provide tentative support for alterations in emotion expressivity serving as a trait vulnerability factor for these disorders.

In anorexia nervosa, there is some evidence for trait related socio-emotional difficulties from retrospective studies which have investigated premorbid temperament characteristics. Although no studies have focussed specifically on premorbid deficits in emotional facial expression, studies have reported negative emotionality and inhibition of emotion [17], impairment in interpersonal functioning [18] and shyness and limited social networks [19] in people who later developed AN. In addition, 20% of individuals with AN report social anxiety before the onset of their eating disorder [20].

Studies with recovered AN individuals also highlight distinct emotional and interpersonal features such as behavioural avoidance, submissive behaviour [21,22] and difficulties recognising emotion in others [23]. However, other studies have shown that recovered AN show no differences to HC in emotion recognition [24]. Therefore, further studies are required to establish whether socio emotional difficulties are present in recovery or can be only observed in the ill state.

In summary, limited studies in AN show that socio-emotional difficulties, which occur in the acute phase of the illness, are present premorbidly, and to some extent following recovery. Many of these studies are using self report questionnaires (e.g. [21,22]) or focus on emotion recognition (e.g.,[23]). Given the importance of emotion expression for effective communication and self regulation, the investigation of whether such behavioural attributes constitute an integral part of the patients' personality and thus contribute to the pathogenesis of AN, is important to develop more accurate and refined illness models.

### Aim

This study employs an experimental method to address whether alterations in emotional facial expression are associated with the acutely ill stage or whether these alterations are present in people recovered from AN. It was hypothesised that people recovered from AN would show some problems with emotional facial expression reflecting the possibility of trait alterations.

## Methods

Ethical approval was obtained through the Oxfordshire Rec NHS ethics committee (reference 08/H0606/58) and the research is in compliance with the Helsinki Declaration.

### Participants

All participants were female and eligible to take part if they were aged between 16 and 55 years old (as the ED unit where recruitment took place treats people in this age range). AN participants were recruited from the South London and Maudsley NHS Foundation Trust’s (SLaM) Eating Disorders Service, through posters placed in the local community and through a circular e-mail to staff and students at King's College, London. For inclusion in this group, participants had to have a BMI (kg/m^2^) below 18.5 and be diagnosed by experienced clinicians as fulfilling DSM-IV (24) criteria for AN. The decision to include this weight limit is based on a recent large meta-analysis which suggests that AN with a more lenient weight criterion and without amenorrhoea is very similar to strict diagnostic AN, as long as BMI < 18.5 is set [25].

Inclusion criteria for the healthy control group included a BMI of between 19 and 25, no personal or family history of psychiatric illness, DSM-IV Axis I disorder or a past or current eating disorder.

For the recovered AN group (RecAN), the structured clinical interview for DSM-IV disorders (SCID) [26] confirmed that participants had previously had AN. Recovery was defined as the absence of eating disorder pathology, together with a BMI of ≥ 19 kg/m^2^ and regular, restored menstruation for at least one year prior to the assessment. Furthermore, recovered participants were required to obtain a score below the clinical cut off of 3 on the Eating Disorders Examination Questionnaire (EDE-Q) [27]. The SCID also determined that the RecAN group did not meet criteria for any current DSM-IV diagnosis.

Exclusion criteria for all groups included poor literacy - as defined by the National Adult Reading Test (NART) [28], non-fluent English, a history of head injury or neurological disease.

Based on the pilot study of Davies et al. [8], the power calculation indicates that a sample size of 15 participants in each group would have 80% power to detect significant differences (d = 1.096) between groups with a 0.05 two-tailed significance level.

### Measures and procedure

All participants gave written, informed consent to take part, after receiving a full explanation of what the study entailed. Self-report questionnaires were completed prior to meeting with the researcher.

### Demographic and clinical information

Participants recorded their age, medication status and ethnicity. Participants’ weight and height was recorded and, in addition, duration of illness and lowest weight was obtained for the recovered and currently ill AN participants. The EDE-Q [27], and Depression, Anxiety and Stress Scale (DASS) [29], were completed by participants.

### Experimental task – films task

Film clips have previously been used by us and other groups as a means to elicit facial emotion expression e.g. [8,30]. Films were chosen as an ecologically valid method for eliciting emotion and do not rely on participants’ ability to recall past experiences, and unlike slides or photographs, provide a more realistic context in which emotional experiences typically develop over time. Three film clips were used, a positive film clip taken from “Four Weddings and a Funeral”, a negative film clip from “Shadowlands” and a neutral film clip, of simulated waves. The positive and negative film clips were selected from a total of 11 after being viewed by 20 people who met HC criteria. The film clips were selected based on eliciting the highest positive and negative scores on the PANAS. Each clip lasts 2 min. Films were presented in a fixed order of positive, neutral, and negative. This is based on the premise that negative affect has a more lasting carry over effect. Participants viewed the clips on the same day and they were presented on a 15-inch computer screen.

### Procedure

While viewing each film clip, participants’ faces were recorded with their knowledge and agreement, using a small camera fixed onto the computer screen. Participants were instructed to “allow themselves to get into the story” as much as possible. This line of instruction was intended to allow participants to attend to the film without revealing the true nature of the study in order to reduce demand characteristics [31]. Following the positive and negative film clip, participants completed the positive and negative affect scale (PANAS) [32]. Because the neutral film clip was primarily included to minimize affect and thus reduce carry over effects between the positive and negative film clips, PANAS ratings were not obtained after the showing of this clip.

### Coding the facial expressions

Recordings of participants were coded using the Facial Expression Coding System (FACES) [33]. In FACES, an expression is defined as any change in the face from a neutral display (i.e. no expression) to a non-neutral display and back to a neutral display. If the participant shifts from neutral to non-neutral but does not return to a neutral display but evidences another clear display of affective expression, this is coded as an additional expression. Each time a separate expression occurs, coders’ rate whether it is a positive or negative expression (valence) and a frequency count of expression is initiated. The intensity of the expression is also assessed using a 4-point Likert scale (1 = low, 4 = very high) as is duration (in seconds). In addition, the frequency of “looking away” is also counted. At the end of each clip, coders compute the following information (1) the total number (frequency) of positive expressions, their mean intensity, and mean duration; (2) the total number of negative expressions, their mean intensity, and mean duration; (3) the total number of times looking away. Two researchers rated the participants’ facial expressions. Inter-rater agreement between coders was medium size (k = .63), which is regarded as substantial agreement [34].

### Analysis

Normality of data was assessed using the Kolmogorov-Smirnoff test and inspection of histograms. Not all variables in the experimental data met parametric assumptions. Data which were positively skewed were log transformed. Experimental data were then analysed using a repeated measures design with planned pairwise comparisons. Where the demographic and clinical data did not meet parametric assumptions, non-parametric Kruskal-Wallis alternatives were used with Mann Whitney U tests to compare groups at the post hoc level. Cohen’s *d* effect sizes were calculated using means and standard deviations and were defined as small (d ≤ 0.4), medium (d > 0.4) or large (d > 0.8). Non-parametric Spearman rank correlation coefficient investigated the relationships between task performance and demographics and clinical symptomatology. Correlations were conducted at the group level.

## Results

Table 1 shows the final study sample which consisted of 123 participants (49 AN, 21 RecAN and 53 HC). The AN participants form an extended group who were reported in a previous study [8].

**Table 1:** Demographic and clinical features for AN, RecAN and HC participants

	**AN (n=49) M (SD)**	**RecAN (n=21) M (SD)**	**HC (n=53) M (SD)**	**Test statistic**	**p value**	**Post hoc difference**	**Cohen’s d ES**
Age	25.9 (6.8)	28.4 (8.7)	26.4 (8.4)	X^2^ = 1.3	n/s	p>0.01	n/a
NART	109.2(8.1)	112.4 (7.5)	113.7(5.9)	F = 10.5	n/s	**AN=HC**	0.91
						**AN=RecAN**	0.66
						**RecAN=HC**	0.20
BMI	14.7 (1.9)	21.0 (2.2)	21.6 (1.6)	F =01.5	<.001	AN<HC**	3.92
						AN<RecAN**	3.06
						**RecAN=HC**	0.31
Illness length	10.1 (6.3)	5.5 (3.5)	n/a	U=204.5	<.001	AN>RecAN**	0.90
Lowest BMI	14.4 (2.8)^b^	13.6 (2.1)^c^	n/a	U=142.0	n/s	p>0.01	n/a
^a^EDE-Q	3.8 (1.7)	1.5 (1.0)	0.6 (0.6)	X^2^=64.5	<.001	AN>HC**	2.51
						AN>RecAN**	1.64
						RecAN>HC*	1.09
^a^DASS anxiety	21.4(10.9)	5.7 (6.5)	2.2 (3.2)	X^2^=66.0	<.001	AN>HC**	2.39
						AN>RecAN**	1.74
						RecAN>HC*	0.68
^a^DASS depression	27.2(11.3)	8.4 (7.7)	2.4 (2.6)	X^2^=72.9	<.001	AN>HC**	3.02
						AN>RecAN**	2.00
						RecAN>HC*	1.04

M=Means; SD=standard deviation; AN = anorexia nervosa; RecAN = recovered AN; HC = healthy controls; NART National Adult Reading Test; BMI Body Mass Index; EDE-Q Eating Disorders Examination; DASS Depression, Anxiety and Stress Scale – HC were excluded if scoring above 9 for depression and 7 for anxiety; ES = Effect Size; U = Mann Whitney U Test; X2 = Kruskal Wallis Test F = Analysis of Variance Test a = 6 AN missing, 3 HC missing; b= 23 missing, c= 8 missing; *comparison significant at p<0.01 level; **comparison significant at p<0.001 level, ns = non-significant.

### Demographic and clinical outcomes

Of the RecAN group, 57% were recruited from King’s College London and the remainder from the community. Of the AN participants, 74% were inpatients, 14% outpatients and 12% recruited from the largest UK charity for eating disorders B-eat.

As illustrated in Table 1, all three groups were comparable in age and IQ. The RecAN group were comparable in BMI to the HC group which was significantly higher than the AN participants. The AN group had a significantly longer duration of illness (10.9 years) than the RecAN (5.5 years), however, there was no significant difference in each of the groups’ lowest BMI since the onset of illness. In terms of clinical variables, as expected the AN group scored significantly higher in eating pathology, depression, anxiety and obsessive compulsiveness than the HC and RecAN groups. The RecAN group scored significantly higher than the HC group on these measures, however, their scores were not in the clinical range. Concerning medication, 39% of the AN group were taking psychotropic medication as were 19% of the RecAN group.

### Experimental paradigm outcomes

There was an overall high correlation between each of the three dependent variables (frequency, intensity and duration) for positive and negative expression. Because of this, and like other studies that have used the FACES system (e.g.[31]) we used “frequency” as the prime index of emotional expressivity in order to reduce the number of highly correlated dependent variables.

### Frequency of emotional expressivity

A three (AN vs RecAN vs HC) x three (amusing, sad, neutral) repeated measures ANOVA was conducted separately for frequency of positive and negative expressiveness, with group as a between subjects factor and film type as a within subjects factor. Table 2 summarises mean scores, standard deviations and effect sizes for each of the experimental variables.

**Table 2:** Facial expression and PANAS response to film clips for AN, RecAN and HC participants

	**Amusing film clip**					**Sad film clip**					**Neutral film clip**			
	**AN (n=49) M (SD)**	**RecAN (n=21) M (SD)**	**HC (n=53) M (SD)**	**post hoc differences**	**Cohen’s *d *** **ES**	**AN (n=49) M (SD)**	**RecAN (n=21) M (SD)**	**HC (n=53) M (SD)**	**post hoc differences**	**Cohen’s *d *** **ES**	**AN (n=49) M (SD)**	**RecAN (n=21) M (SD)**	**HC (n=53) M (SD)**	**post hoc**
Positive expression^1^	4.0	8.6	11.0	AN<HC**	**1.72**	0.1	0.2	0.3	p>0.01		0	0	0	p>0.01
	(4.1)	(4.2)	(4.0)	AN<RecAN**	**1.10**	(0.3)	(0.5)	(0.8)			(0)	(0)	(0.2)	
				RecAN=HC	0.50									
Negative expression^1^	0.8	1.0	0.2	AN>HC*	**0.59**	0.8	1.6	2.9	AN<HC**	**1.13**	0	0	0	p>0.01
	(1.3)	(1.3)	(0.7)	AN=RecAN	0.15	(1.4)	(2.0)	(2.2)	AN=RecAN	0.46	(0.2)	(0.2)	(0.2)	
				RecAN>HC*	**0.74**				RecAN=HC	0.61				
Looking away^1^	0.9	0.2	0.5	p>0.01		4.6	1.6	0.6	AN>HC**	**1.11**	2.6	1.7	3.2	p>0.01
	(2.0)	(0.5)	(1.9)			(5.0)	(2.5)	(1.0)	AN>RecAN**	**0.75**	(4.6)	(2.1)	(6.5)	
									RecAN=HC	0.52				
PANAS positive^2^	11.2	13.8	16.9	AN<HC**	**0.86**	4.6	7.0	7.1	AN<HC*	**0.52**	-	-	-	
	(6.7)	(6.0)	(6.5)	AN=RecAN	0.40	(4.4)	(6.6)	(4.6)	AN=RecAN	0.42				
				RecAN=HC	0.50				RecAN=HC	0.01				
PANAS negative^2^	3.0	1.1	1.0	p>0.01		7.6	7.1	6.5	P>0.01		-	-	-	
	(4.0)	(2.0)	(2.3)			(7.3)	(5.1)	(4.8)						

M = Means; SD = standard deviations presented in brackets; AN = anorexia nervosa; RecAN=recovered anorexia nervosa; HC = healthy controls; PANAS = positive and negative affect scale; ES= effect size; ^1^frequency count; ^2^PANAS not recorded subsequent to neutral film clip; * = p <.01, ** = p <.001.

### Positive expression

For positive expressivity the data met parametric assumptions. There was a significant main effect for film type [F(2, 240) = 379.8, p < .001, partial ϵ^2^ 0.8] and for group [F(2,120) = 37.9, p < .001, partial ϵ^2^ 0.38]. The response pattern to film type differed between groups as the significant film type x group interaction revealed [F(4,240) = 37.0, p < .001, partial ϵ^2^ 0.4]. Planned pairwise comparisons showed there was a significant difference in the frequency of positive expression in response to the amusing film between AN and HC groups (mean difference 7.0, 95% confidence interval [CI] 5.3 to 8.6, p < .001, d = 1.72) with HC showing higher frequency of positive expressions. Also between the AN and RecAN groups (mean difference 4.6, 95% CI 2.5 to 6.7, p < .001, d = 1.10) with RecAN showing more positive expressions. There was no significant difference between the RecAN and HC groups (p = .12).

In response to the sad and neutral film clips there was no significant difference in positive expression between the groups as p >0.01.

### Negative expression

Data were log transformed due to non normal distribution and unequal variances. There were significant main effects for film type [F(2, 240) = 68.8, p < .001, partial ϵ^2^ 0.4] and for group [F(2, 120) = 158.7, p < .05, partial ϵ^2^ .05]. The response pattern to film type between groups differed as the significant film type x group interaction showed [F(4, 240) = 15.4, p < .001, partial ϵ^2^ 0.2]. A planned pairwise comparison showed there was a significant difference in frequency of negative expression in response to the sad film clip between the AN and HC groups (mean difference 0.27, 95% CI 0.2 to 0.4, p < .001, d = 1.1) with the AN group showing less negative expression to the sad film clip than the HC group. There was no significant difference between RecAN and HC (p = 0.7).

In response to the positive film clip, negative expression significantly differed between the AN and HC groups (mean difference 0.12, 95% CI 0.4 to 2.0, p < .004, d = 0.5) and between the RecAN and HC groups (mean difference 0.12, 95% CI 0.07 to 0.3, p < .001, d = 0.7) with the AN participants and RecAN group showing higher frequency of negative expression towards the amusing film clip than the HC group.

There was no difference in negative expression in response to the neutral film clip between groups.

### Looking away

A significant main effect was found for film type [F(2,240) = 8.7, p < .001, partial ϵ^2^ .07] and for group [F(2,120) = 4.2, p = .02, partial ϵ^2^ .07] and a significant interaction between filmtype x group [F(4, 240) = 74.9, p < .001, partial ϵ^2^ .10]. Planned pairwise comparisons showed no significant difference between groups in looking away from the amusing film clip but there were in response to the sad film clip between the AN and RecAN groups (mean difference 3.0, 95% CI 1.3 to 4.8, p < .001, d = 0.7) and between the AN and HC groups (mean difference 4.0, 95% CI 2.6 to 5.3, p < .001, d = 1.1) with the AN group looking away more from the sad film clip than the RecAN and HC groups. There was no significant difference between the RecAN and HC in looking away from the sad film clip (p = .12).

There were no significant differences in frequency of looking away from the neutral film clip as p > 0.01.

### Emotional experience

#### Positive affect (PANAS)

There were significant main effects for filmtype [F(1,119) = 188.7, p < .001, partial ϵ^2^ 0.6] and for group [F(2,119) = 8.7, p < .001, ϵ^2^ 0.1] and a significant interaction between filmtype and group [F(2,119) = 4.6, p < .01, partial ϵ^2^ .10]. Planned pairwise comparisons showed there was a significant difference in subjective positive response to the amusing film clip between the AN and HC groups (mean difference 5.6, 95% CI 3.1 to 8.2, p < .001, d = 0.8) with the HC reporting higher positive affect. There was no significant difference between the RecAN and AN groups (p = .14, d = 0.3) or RecAN and HC group (p = 0.6, d = 0.5). Regarding positive subjective response towards the sad film clip there was a significant difference between the HC and AN participants (mean difference 2.5, 95% CI 0.6 to 4.3, p < .01, d = 0.5) with the HC group reporting a higher level of positive affect towards the sad film clip. There was no significant difference between the RecAN and HC groups.

#### Negative affect (PANAS)

There was a significant main effect for filmtype [F(1, 119) = 198.2, p < .001] but not for group [F(2, 119) = 2.0, p = .14]. Therefore, no further planned pairwise comparisons were conducted.

#### Assessing age as a confound

Although there was not a significant difference in age between groups, mean age was higher in the RecAN group. Expressivity has been shown to decrease with age, therefore, it was deemed appropriate to run the repeated measures analysis including age as a covariate. Age did not significantly contribute to the significant main effect for group for positive expression [F(1, 119) = 0.0, p = .99] or for negative expression (F(1,119) = 0.28 p = .60) and the main effect between groups remained significant.

#### Medication

The analyses for the experimental outcomes were run comparing medicated to un-medicated participants. Nineteen people in the AN group were taking antidepressants. There were no significant differences between medicated and un-medicated people in frequency of positive expression (p = .46), negative expression (p = .44), looking away (p = .38), subjective positive affect (p = .53) or negative subjective affect (p = .92). Four people in the RecAN group were taking antidepressants. There were no significant differences between medicated and un-medicated people in frequency of positive expression (p = .32), negative expression (p = .22), looking away (.89), positive affect (.08) or negative affect (.54). Therefore, expressive alteration does not appear to be a function of medication status.

#### Correlational analysis

##### Relationships between facial expression and clinical variables

Correlations were run within each group and the only significant relationships were found within the AN group. Positive facial expression was negatively related to depression (r_s_ = −.30, p < .001) and anxiety and depression were negatively related to positive expression to the sad film (r_s_ = −.40, p < .001), (r_s_ = −.47, p < .001), respectively. This suggests that the higher the depression the lower the display of positive facial expression to both the amusing and sad film clip. No relationships were found in the RecAN group between clinical variables and facial expression.

##### Relationships between subjective emotion and clinical variables

In the AN group there were positive moderate size correlations between negative subjective affect to the amusing film clip and anxiety (r_s_ = .45) and depression (r_s_ = .54). This is interpreted as the more clinically symptomatic the less positive emotion is felt in response to the amusing film clip. All correlations are significant at the p < .001 level.

## Discussion

We have previously shown altered emotional facial expression in people currently ill with AN [8]. The present study aimed to examine whether these alterations exist in individuals recovered from the illness. The key finding was that in response to the amusing film clip RecAN participants showed as much positive, but a higher frequency of negative expression (i.e. similar to the currently ill group) compared to the HC group^a^.

There was no difference in response to the sad film clip between the RecAN and HC, whereas the currently ill group showed less facial expression and looked away more from the sad film clip. It could be hypothesised, however, that the group showed less negative facial expression because they looked away more from the film clip.

Alterations shown in the AN group in response to the sad film clip, such as looking away, could be construed as an attempt to avoid any negative feelings the stimulus was evoking (see Davies et al., 2011 for further discussion on this). This is supported by previous studies, which suggest that avoidance in AN is used as a means of reducing affective states [35]. It could be speculated that avoidance of negative emotion could be a relevant factor as regards treatment resistance.

The RecAN responses to the amusing film clip are interesting, and could be interpreted in a number of ways. First, the theme of the amusing film clip, a wedding, may just be more aversive for the AN and RecAN groups. Due to longevity of illness and missed opportunities in relationships, the clip may elicit more negative feelings and behaviours. Future studies would need to use different film clips involving a range of emotions to ascertain whether negative expressions pertain to other positive stimuli. Nonetheless, it should be noted that other studies with currently ill AN groups have shown negative facial activity to appetitive stimuli, measured using electromyography and startle response [36,37].

Another potential explanation for an increase in negative facial expression to the amusing stimuli, is related to problems with facial mimicry. There is tentative evidence for emotion recognition difficulties in currently ill and RecAN individuals, with both groups showing a significantly higher social and angry-threat attentional bias [7]. Facial mimicry of emotion is the visible or non-visible use of facial musculature by an observer to match the facial gestures in another person’s expression [38], this often occurs at an automatic and unconscious level and helps to synchronise emotion and thus empathy between people [39]. It could be that due to a misinterpretation and bias towards negative facial displays, facial responding is in turn inaccurate borne out in increased negative expression.

Finally, it is possible that ‘scarring’ effects are present in people who are recovered from AN [40] and contribute to the alterations highlighted. As the RecAN group showed a mean illness duration time of five years, scarring could be apparent with incomplete recovery of emotion expression. Longitudinal studies which assess at risk cohorts premorbidly and compare those who do or do not develop the disorder would be a more accurate way to examine these alterations and address the state/trait nature of facial expressivity to emotional stimulus.

Interestingly, the RecAN did show a similar frequency of positive expression to the amusing film as the HC (whereas the currently ill group showed less). It may be possible, however, that these expressions were not ‘authentic’. In the facial expression literature there is a distinction between involuntary ‘authentic’ expressions and voluntary ‘inauthentic’ expressions [41], with studies showing that ‘authentic’ expressions lead to better social interaction [12]. The RecAN subjective reports of positive affect (measured by the PANAS) showed an intermediate profile between currently ill and HC groups (medium effect size) and could suggest ‘strategic’ as opposed to hedonic behavioural responses to amusing stimuli. It would be beneficial to understand if recovered individuals use more cognitive ‘strategic’ voluntary displays of positive expression, especially as social maladjustment has been suggested to still be present after recovery e.g. social anhedonia [42,43] and with impairment in interpersonal functioning in people who later developed AN [18]. Coding systems such as the Emotion Facial Action Coding System (EMFACS) [44] which make the distinction between authentic and non authentic positive expressions (based on muscle movement) should be employed in future studies.

### Limitations

One of the problems with a cross sectional design is the comparability of groups. For example, the RecAN group may not originally have been as ill as the currently ill group. Although reported lowest weights for RecAN were as low as the AN group (albeit approximately half of this data was missing), illness length for RecAN was shorter and it is not known if their clinical symptomatology was as severe. Also, by the sheer fact of having recovered, these individuals may be a good prognostic group, with lower levels of risk or maintaining factors for AN. In contrast, 74% of the AN group were recruited from an inpatient unit and thus were people at the severe and enduring end of the clinical spectrum, i.e. may be a poor prognostic group. Therefore, the RecAN group premorbidly may have shown less alteration in facial expression and be a positive factor in terms of resistance to treatments i.e. they show less resistance to treatment as they have better social interaction and emotion regulation skills.

Another limitation is that 19% of the recovered group were taking antidepressant medication, raising the question of how well recovered they were in terms of affective symptoms.

Finally, the sample size for the recovered group was small which may have biased findings and limits our statistical power and conclusions.

## Conclusion

This study shows that alterations in emotional facial expression observed in people currently ill with AN are not as far reaching in a recovered group, however, minor alterations are present. This is in keeping with observations of personality changes and diminished desire for social interaction and positive reciprocity in the starved state and at low weight as well as a general resolution following resumption of normal eating and weight gain [13]. Alterations in facial expression in people currently ill with AN, compared to HC, contribute to the social and emotional difficulties shown in the illness and potentially play a role in resistance to treatment.

The negative expression to amusing stimuli observed in the RecAN group needs to be scrutinised further to see whether it is a behaviour which manifests in response to different positive stimuli. Different emotion film clips could be used for this along with a coding system such as EMFACs [44] which codes for discrete emotions and detects differences between voluntary and non voluntary facial expressions which would be helpful in answering questions about strategic versus genuine positive responses. Finally, a longitudinal study assessing facial emotion expression in patients when they are ill and when they are recovered would be a reasonable next step.

### Endnotes

^a^N.B. In the previous study (Davies et al., 2011) the currently ill AN patients did not show significantly more negative emotional expression during the positive film clips compared to HC. This discrepancy may be explained by the larger sample size in the current study (even more so as there is a trend towards a significant difference between AN and HC in the previous study (p value = .06).

## Abbreviations

AN: Anorexia nervosa; RecAN: Recovered anorexia nervosa; HC: Healthy control; BMI: Body mass index; PANAS: Positive and negative affect scale; PTSD: Post traumatic stress disorder; FACES: Facial expression coding system; EMFACS: Emotional facial action coding system; ANOVA: Analysis of variance.

## Competing interests

The authors declare that they have no competing interests.

## Authors’ contributions

HD contributed to the conception and design, acquisition, analysis and interpretation of data and drafting manuscript; US and KT contributed in the conception and design of the study, interpretation of the results, drafting and editing the manuscript. All authors read and approved the final manuscript.

## Pre-publication history

The pre-publication history for this paper can be accessed here:

http://www.biomedcentral.com/1471-244X/13/291/prepub
